# Comparing and Correlating Outcomes between Open and Percutaneous Access in Endovascular Aneurysm Repair in Aortic Aneurysms Using a Retrospective Cohort Study Design

**DOI:** 10.1155/2020/8823039

**Published:** 2020-11-27

**Authors:** Peter DeVito, Ali Kimyaghalam, Sameh Shoukry, Robert DeVito, John Williams, Eashaa Kumar, Eugene Vitvitsky

**Affiliations:** ^1^University of Arizona, Tucson, AZ, USA; ^2^Department of Vascular Surgery, Trumbull Regional Medical Center, Warren, OH, USA; ^3^American University of Antigua College of Medicine, Coolidge, Antigua, USA; ^4^Northeast Ohio Medical University, Rootstown, OH, USA

## Abstract

**Objective:**

This retrospective cohort study is aimed at determining the safety and efficacy between Femoral Open-Cutdown access and Percutaneous access with Endovascular Aneurysm Repair (EVAR) by contrasting perioperative complication rates. We hypothesized that the percutaneous approach is a better alternative for aortic aneurysm patients as it is minimally invasive and has been demonstrated to decrease the length of hospital stay.

**Methods:**

We retrospectively reviewed data for patients undergoing EVAR between the years of 2005 and 2013. We then compared overall mortality, hematoma or seroma formation, graft infection, arterio-venous injury, distal embolization, limb loss, myocardial infarction or arrhythmia, and renal dysfunction. Results were demonstrated using a retrospective cohort study design to confirm the hematoma rate associated with EVAR open compared to percutaneous access.

**Results:**

Our series involves 73 patients who underwent percutaneous access for EVAR (*n* = 49) or traditional open cutdown (*n* = 24). Percutaneous access resulted in significantly less hematoma formation when compared to the traditional open cutdown (4% vs. 12.5%; *p* < 0.059). Our analysis suggests decreased mortality rates associated with EVAR as compared to the Open-Cutdown method using Northside Medical Center's Study and the OVER Veterans Affairs Cooperative Study (*p* = 0.0053).

**Conclusion:**

Percutaneous access for EVAR is safe and effective when compared to Open-Cutdown access for aortic aneurysm patients. Percutaneous access was associated with decreased rates of in-hospital mortality, hematoma formation, graft infection, and respiratory failure.

## 1. Introduction

Aortic aneurysms have a well-established correlation with hypertension and atherosclerotic disease, and the treatment has evolved over the years. The advancement in surgical techniques to deliver grafts has vastly transformed the perception of aortic surgery. Endovascular Aneurysm Repair (EVAR) has essentially overtaken open repair of aortic aneurysms mainly due to the minimally invasive nature of EVAR as well as the decreased length of hospital stay [1]. The femoral artery is typically the vessel used for access during EVAR and access to the femoral vessels has generally been broken down into two categories: Open-Cutdown and Percutaneous access [2]. An Open-Cutdown method is a traditional technique of fully exposing the femoral vessels and subsequently closing the site primarily. The percutaneous method places the suture-mediated closure device at the beginning of the procedure before inserting the large bore sheaths.

While the decision to perform traditional Open-Cutdown access or Percutaneous access is primarily due to physician preference, few large-scale studies evaluate the differences in the complication rates of both methods of access [3, 4]. Through a case-based approach, single institutions review complications of EVAR access and demonstrate these findings in current studies [5].

A study performed by Cao and colleagues showed that Percutaneous access was associated with fewer complications, shorter hospital length of stay, and shorter procedure time [3]. One of the first multicenter trials comparing Percutaneous EVAR with open femoral EVAR was the study by Nelson and colleagues, which showed the Percutaneous approach to be noninferior to the open femoral approach for complications such as blood loss, groin pain, and quality of life [6]. Despite a limited sample size of 150 patients, this multicenter randomized clinical trial provides valuable information about the Percutaneous approach versus EVAR.

Physicians at our institution prefer to use the Percutaneous approach to femoral access for EVAR. This study intends to combine data from our institution with the data from prior studies to compare perioperative complication rates between Femoral Open-Cutdown access and Percutaneous access for EVAR with the hope of showing that the Percutaneous approach is an equally safe, or possibly better, alternative.

## 2. Methods

We retrospectively reviewed data from Northside Medical Center and searched for patients undergoing EVAR between the years of 2005 and 2013, with most patients undergoing Percutaneous access and a small percentage having the open femoral cut-down approach. Complication rates that we compared included overall mortality, graft infection, hematoma formation, limb loss, renal impairment, myocardial infarction or arrhythmia postoperatively, and distal embolization. Each complication was diagnosed with the respective “gold-standard” test, such as using ultrasound for hematomas, to ensure accuracy. Immediate in-house perioperative complications and at a 30 day follow-up after discharge were evaluated. Our case series involves 73 patients. Of these, 49 underwent Percutaneous access for EVAR, and 24 underwent the traditional open cutdown. We compared overall mortality, hematoma or seroma formation, graft infection, arterio-venous injury, distal embolization, limb loss, the incidence of myocardial infarction or arrhythmia postoperatively, and associated renal dysfunction. We then used our statistical analysis to solidify the results of the hematoma rate associated with EVAR. Outcomes are provided in Table 1. This provides perspective when compared with the open repair rate of hematoma when considering the Northside Medical Center Study results. We then used an analysis that incorporated figures for hematoma formation from both Northside Medical Center as well as the Cleveland Clinic Trial [7] to increase the statistical power and significance regarding the rate of hematoma in EVAR in Table 2. Another meta-analysis was performed to confirm a decreased mortality rate associated with EVAR as compared to the Open-Cutdown method using the figures from the Northside Medical Center Study and OVER study from Veterans Affairs Cooperative Study in Table 3, yielding a statistically significant correlation [8].

## 3. Results

Our results shown in Table 1 indicate that Percutaneous access resulted in less hematoma formation when compared to the traditional open cutdown (4% vs. 12.5%; *p* < 0.059). One patient developed distal embolization (to the distal SFA) in the Open-Cutdown group, and two patients in the Percutaneous group developed similar results. In the Open-Cutdown group, one patient developed postoperative atrial fibrillation, and one patient developed renal dysfunction marked by decreased urine output and a rise in serum creatinine.

One patient of forty-nine Percutaneous access patients experienced in-hospital mortality. This was an emergent ruptured EVAR, and the patient suffered severe hemodynamic instability due to exsanguinating retroperitoneal hemorrhage. Three patients in the Percutaneous group developed postoperative atrial fibrillation, and one patient had postoperative renal dysfunction.

Analysis verifies the statistical significance of our associated risk of hematoma associated with EVAR as compared to Cleveland Clinic's study of associated outcomes with EVAR [7] (*p* = 0.0285). Separate analysis suggests that there may be decreased mortality rates associated with EVAR as compared to the Open-Cutdown method using Northside Medical Center's Study and the OVER Veterans Affairs Cooperative Study (*p* = 0.0053) (Table 1).

## 4. Discussion

Since 1991 [9], EVAR has been cultivated to become one of the most widely used techniques in the treatment of abdominal aortic aneurysms (AAA) [10]. The safety and efficacy of EVAR have historically addressed the risks of open surgery and anesthesia, patient discomfort, length of hospital stay, and faster return to daily activities of life [11]. Approximately 80% of all EVAR procedures in the United States continue to be performed with general or spinal anesthesia, along with open surgical femoral arterial access and repair [12]. In this study, the authors conducted a Retrospective Case Series analysis evaluating the effectiveness of the Percutaneous closure of the femoral artery to an Open-Cutdown technique with surgical exposure and control of the common femoral arteries. The authors then performed two analyses on the occurrence of hematoma including Northside Medical Center and OVER study and the mortality rate associated with EVAR including Northside Medical Center and OVER study [7].

Current literature suggests that Percutaneous access to the femoral artery with ultrasound guidance is more advantageous than open surgical repair in the management of aortic aneurysms [3, 4, 13]. In a retrospective study collecting prospective clinical data from the American College of Surgeons National Surgical Quality Improvement Program (ACS NSQIP) database, Buck et al. identified 3004 patients with open surgical repair and 1108 patients undergoing Percutaneous access [4]. Study findings were significant for a technical success rate of 96% in the Percutaneous EVAR population and demonstrated significantly shorter hospital length of stay (LOS), shorter operation time, and fewer wound complications [3–5, 14].

Disadvantages observed in the Percutaneous method include distal embolization as well as pseudoaneurysms [3]. Other factors to consider affecting the complexity of Percutaneous EVAR access include potential life-long postprocedural surveillance [15] and comorbidities which may predispose the patients to complications, such as small vessel diameter and vascular calcifications [3, 4, 16, 17]. Regarding the Open-Cutdown method, not only has this technique been noted to increase invasiveness but also it has been observed to endorse complications such as post-op hematoma [5, 12].

A fundamental objective to address is the incidence of forming large incisions in the groin with associated complications [6]. The Percutaneous technique allows us to perform the same intricate procedure in a much more minimally invasive way, with decreased surgical site complications and using a local anesthetic. This could prove to be of great value in emergent cases where hemodynamic instability precludes the use of general anesthetic. Understandably, some may be leery of the idea of Percutaneously closing large femoral arterial holes, but with the advent of Percutaneous closure technologies, we can be assured that in experienced hands this technique can lead to fewer complications when compared to the traditional approach.

Our study showed precisely this that there is a lower rate of complications and mortality associated with percutaneous EVAR as compared to the Open-Cutdown procedure.

## 5. Study Limitations

The study limitations are as follows:

- Retrospective (NS study)
- Limited sample population
- Localized to one hospital
- Selection bias
- Publication bias
- Not standardized
- Theoretical relationship instead of a causal association
- The analysis includes two studies; the number of studies needs to be increased to decrease the bias further

Further studies should be conducted evaluating set parameters to gather reliable data comparing open repair with EVAR surgical technique comprehensively:

- We would like to do a larger meta-analysis on the data that is published and combined our data with the results from other multiple studies
- We would like to suggest that variables be made common among larger studies to enable a more integrated meta-analysis specifically analyzing hematoma, incision site infection, and other parameters that allow for broader comparison among numerous studies
- We would like to conduct a large-scale, prospective study, over a 5- to10-year period

## 6. Conclusion

Overall, we found that there is a lower mortality rate associated with percutaneous EVAR as compared to the Open-Cutdown procedure, which is verified by analysis of the Northside Medical Center Study and the OVER study (Table 3). The Northside Medical Center Study also demonstrated a decrease in the number of hematomas associated with EVAR as compared to the Open-Cutdown procedure, as shown in our analysis, although the result was marginally insignificant likely due to low power. We suggest that further studies be done with similar parameters to obtain more data for statistically significant information on critical factors such as infection at the incision site, hematoma formation, and mortality with EVAR vs. Open-Cutdown method. These parameters should be communicated among leading academic institutions before designing new research studies to ensure that verifiable data is obtained, and patient care is optimized.

## Figures and Tables

**Table 1:** Northside Hospital Study: retrospective EVAR vs. Open-Cutdown method and associated outcome.

Complication	Open Cutdown	Percutaneous	Pearson chi-squared (*p* value)^∗^	Fisher's exact test (*p* value)^∗^
In-hospital mortality	3.1%	1.2%	0.486	0.484
30-day hospital mortality	0%	0%	^∗∗^	^∗∗^
Endograft leak	19.3%	34.6%	0.117	0.168
Stenosis graft	0%	2.5%	0.367	1.000
Ischemic bowel	3.1%	0%	0.110	0.283
Hematoma^∗∗∗^	15.6%	4.9%	0.059	0.071
Seroma	0%	1.2%	0.528	0.717
Graft infection	3.1%	0%	0.110	0.283
Gallbladder complication	3.1%	1.2%	0.492	0.488
AV injury	0%	1.2%	0.528	0.717
Distal embolization	3.1%	3.7%	0.881	0.682
Limb loss	0%	0%	^∗∗^	^∗∗^
Myocardial infarction	3.1%	9.8%	0.238	0.221
Renal dysfunction	12.5%	21.0%	0.296	0.222
Respiratory failure	15.6%	7.4%	0.184	0.164
Thrombocytopenia	21.9%	20.0%	0.917	1.000

^∗^Significant value indicated with *p* value <0.05. ^∗∗^No statistics computed because complication is a constant. ^∗∗∗^Borderline significant with *p* value very close to 0.05, denoting that Open-Cutdown procedure likely to observe hematoma when compared to Percutaneous procedure.

**Table 2:** Analysis of hematoma associated with EVAR. Northside Hospital Study with Cleveland Clinic Study (Sampram et al.).

Variable in common	% of Cleveland Clinic	Number of people % represents	% of Northside Hospital	% of people % represents	Total number of people in group Cleveland Clinic	Total number of people in group NS
Hematoma outcomes in endovascular repair patients	1.1	8	4.9	4.02	703	82
Total # of patients in endovascular groups	703 (CC) + 82 (NS)					
Total endovascular repair meta-analysis group	785					
Meta-analysis hematoma in endovascular repair	(8 + 4.2)/785					
% hematoma in endovascular repair meta-analysis	1.5%					

**Table 3:** Analysis of mortality with EVAR vs Open-Cutdown method: Northside Hospital Study with Open Versus Endovascular Repair (OVER) Veterans Affairs Cooperative Study Group.

Variable in common	% of veterans study	Number of people % represents	% Northside hospital	# of people % represents	Total number of people in group vets	Total number of people in group NS
Mortality endovascular repair	0.5	2.2	1.2	0.984	444	82
Open repair	3	13.11	3.1	0.99	437	32
Total # of pts in endovascular groups	444 (vets) + 82 (NS)					
Total endovascular repair	444 + 82 = 526					
Meta-analysis mortality in endovascular repair	(2.2 + 0.984)/526					
% mortality in endovascular repair meta-analysis	0.6%					

## Data Availability

The data will be available at request. All the data will be included in the excel sheet.
